# A typology of physician input approaches to using AI chatbots for clinical decision-making

**DOI:** 10.1038/s41746-025-02184-y

**Published:** 2025-12-05

**Authors:** Rachel Siden, Hannah Kerman, Robert J. Gallo, Joséphine A. Cool, Jason Hom, Ethan Goh, Neera Ahuja, Paul Heidenreich, Lisa Shieh, Daniel Yang, Jonathan H. Chen, Adam Rodman, Laura M. Holdsworth

**Affiliations:** 1 Department of Medicine, Stanford University School of Medicine, Palo Alto, CA USA; 2 Division of Hospital Medicine, Beth Israel Deaconess Medical Center, Boston, MA USA; 3 Division of Hospital Medicine, Harvard Medical School, Boston, MA USA; 4 Center for Innovation to Implementation, VA Palo Alto Health Care System, Menlo Park, CA USA; 5 Department of Health Policy, Stanford University, Stanford, CA USA; 6 Division of Hospital Medicine, Stanford University, Palo Alto, CA USA; 7 Stanford Center for Biomedical Informatics Research, Stanford University, Stanford, CA USA; 8 Clinical Excellence Research Center, Stanford University, Stanford, CA USA; 9 Kaiser Permanente, Oakland, CA USA

**Keywords:** Health services, Medical research

## Abstract

Recent studies have found that physicians with access to a large language model (LLM) chatbot during clinical reasoning tests may score no better to worse compared to the same chatbot performing alone with an input that included the entire clinical case. This study explores how physicians approach using LLM chatbots during clinical reasoning tasks and whether the amount of clinical case content included in the input affects performance. We conducted semi-structured interviews with U.S. physicians on experiences using an LLM chatbot and developed a typology based on input patterns. We then analyzed physician chat logs from two randomized controlled trials, coding each clinical case to an input approach type. Lastly, we used a linear mixed-effects model to compare the case scores of different input approach types. We identified four input approach types based on patterns of content amount: copy-paster (entire case), selective copy-paster (pieces of a case), summarizer (user-generated case summary), and searcher (short queries). Copy-pasting and searching were utilized most. No single type was associated with scoring higher on clinical cases. Other factors such as different prompting strategies, cognitive engagement, and interpretation of the outputs may have more impact and should be explored in future studies.

## Introduction

Large language model (LLM) chatbots (such as ChatGPT, Perplexity, or Claude) that users can interact with conversationally have proliferated in recent years^1^, including in clinical contexts, and are increasingly being used for generating differential diagnoses^2,3^. The most recent generation of LLM chatbots have demonstrated even greater abilities in addressing complex clinical cases with a high degree of accuracy in controlled settings with researcher-created prompts^4,5^.

The inputs to LLM chatbots can significantly influence the accuracy of outputs produced^6,7^. Prompt engineering, the practice of optimizing LLM chatbot outputs with structured inputs, has emerged as an important field of study^8^. Such inputs can include assigning a role, defining timelines, describing the setting, and providing context^9^, which are additional details that are not typically used with traditional search engines. Prompt engineering best practices are not always intuitive to lay or untrained users, who may employ a variety of different input styles when interacting with LLM chatbots^10,11^. When evaluating LLM chatbot outputs for accuracy and applicability in clinical settings, it will be important to consider not just how LLM chatbots perform under ideal conditions, but also how well they perform with everyday human users. In previous work evaluating the accuracy of chatbot responses to clinical cases, researchers found that a LLM chatbot alone (using structured prompts which contained the entire clinical vignette) scored higher on clinical reasoning cases than physicians working through the same cases with access to the same LLM chatbot^12,13^. It is unknown whether the difference in scoring could be attributed to different styles of inputting content into the LLM chatbot. Specifically, if a key difference was due to how the chatbot was given the entire case vignette, whereas physicians chose for themselves how much content to input.

In this study, we aimed to identify how physicians interacted with an LLM chatbot (GPT-4) in clinical reasoning tasks to create a typology of input approaches, understand the rationales for different input approaches, and explore if any input practices were associated with improved clinical reasoning performance. We hypothesized that more information from the case vignette input into the LLM chatbot would produce more thorough outputs and lead to higher clinical reasoning scores.

**Fig. 1:**
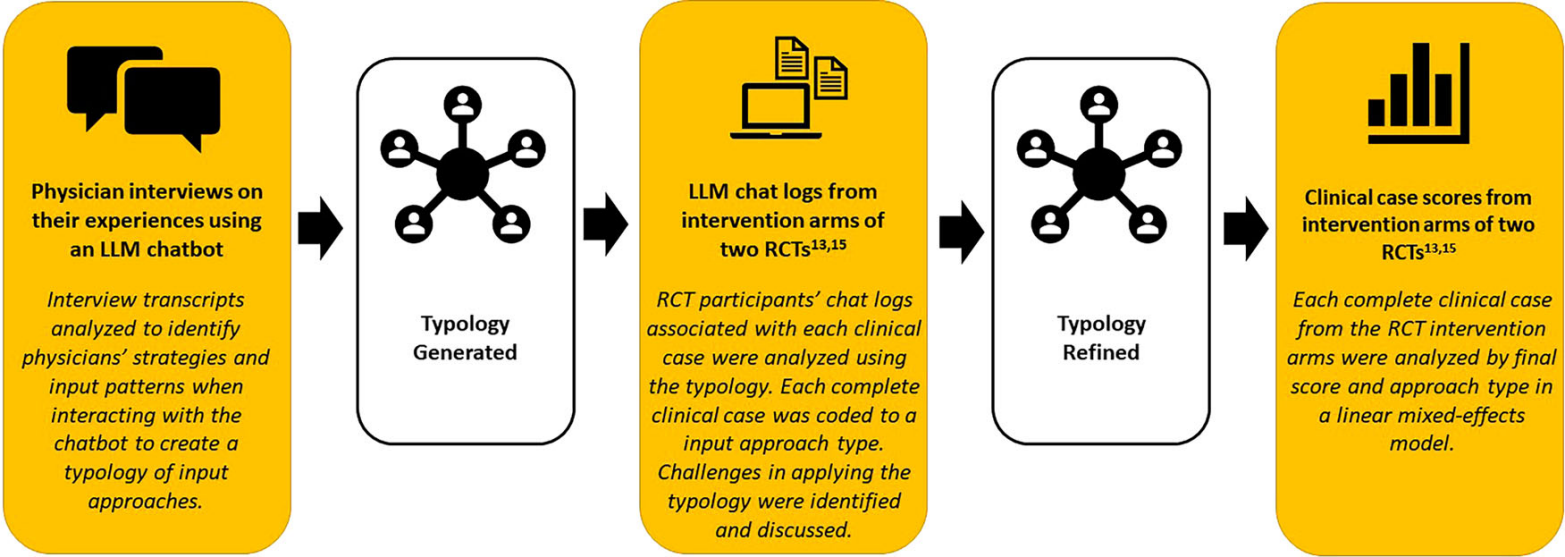
The three research steps in relation to the development of the typology. The yellow boxes indicate the three research steps and the white boxes indicate how the typology was developed. The bolded text in the yellow boxes indicates the type of data that was analyzed and the italicized text describes how the typology was either developed or applied.

We carried out a sequential mixed methods study. A typology approach, which groups participants into types based on common features^14^, was selected to explore different input styles. First, qualitative interviews were conducted with physicians who had completed mock clinical cases using GPT-4 to understand input approaches and generate an initial typology. Next, the typology was used to analyze the recorded interactions (“chat logs”) between GPT-4 and physician participants from the intervention arms of two randomized clinical trials (RCTs)^13,15^, where participants completed diagnostic and management cases using GPT-4. Each clinical case where GPT-4 had been used for problem-solving was coded to an input approach type. Lastly, we then examined whether the input approach types were associated with differences in performance by using a linear mixed-effects model to compare the scores and input approach types of the clinical cases. The study design is visualized in Fig. 1 and each research step is discussed in the methods section.

## Results

We first present the findings from our interviews and the typology development, followed by the analysis of input approach type performance.

### Participant characteristics

Twenty-two physicians were interviewed between May and July 2024; participant characteristics are described in Table 1. Interviews lasted a median of 33 min (range 16–54 min). Physicians reported different experiences using LLM chatbots prior to this study, with most having some experience using LLM chatbots, and six having no or minimal prior experience. All but six physicians described previously using LLM chatbots, with some using them for different reasons (e.g., both personally and professionally). Ten physicians described their LLM chatbot experience as self-taught, where their current knowledge came primarily from reading information online or personal experimentation as opposed to formal training.

**Table 1:** Participant characteristics

Characteristic	*N*
Gender	
Female	12
Male	10
Race/Ethnicity	
White	12
Asian	3
Asian: Indian	3
Hispanic	3
Native American	1
Specialty	
Internal medicine	13
Family medicine	5
Emergency medicine	4
Years of experience	
1–3	15
4–9	5
10+	2
Type of health system	
Academic medical center	16
County hospital or federally qualified health center	4
Veterans affairs	1
Private practice	1
Prior experience with Chatbot AI^a^	
Personal (e.g., vacation planning)	15
Professional (e.g., teaching, coding)	9
Clinical (e.g., differential diagnosis)	7
None or minimal (e.g., “tried it once”)	6

^a^Physicians described multiple uses, therefore *n* > 22.

### Developing and refining the typology

From the interviews, we identified two distinct axes of inputting information into the chatbot. The first axis is “content amount.” Physicians described inputting four different amounts of content from the vignette, from “most” to “least”: copy-pasting the entire vignette, copy-pasting sections of the vignette, summarizing the vignette in their own words, or writing brief queries about topics in the vignette. The second axis is “prompt style” which involved telling the chatbot what to do with the information, such as providing context (e.g., “you are helping a physician solve a clinical case”) or asking it to perform a task (e.g., “provide a differential diagnosis”). In the analysis of chat logs from the RCTs, coders found that the descriptions for “content amount” could be reliably used to identify an input approach. Very few participants used more than one approach within a single case, and all cases were coded to a single type based on the highest amount of content used. However, “prompt style” was difficult to identify in chat logs since physicians may have tried different types of inputs within a single case and it was unclear whether the physician was giving direction to the chatbot. Because prompt style was not clearly identifiable within the chat logs, the final typology was refined to address content amount only, and is described in Table 2.

**Table 2:** Typology of input approaches with descriptions and exemplar quotes

Type	Description	Exemplary quote
Copy-paster	Copies and pastes the whole vignette into the chatbot.	*I basically just inputted the entire vignette and then said, Please list three potential diagnoses based off of the vignette. Physician 14* *If I typed every single thing that I thought was important, that was going to take a lot more time than just me copying everything and just pasting it. Physician 22* *It’s definitely less effort to just copy-paste the whole thing in […] instead of carefully formulating a well-thought-out question. Physician 10* *I think [copy-pasting] was just to give it all the information that we were provided with. I wasn’t sure if there’s a better way to format it. Physician 13*
Selective copy-paster	Copies sections of the vignette, but not the whole thing; makes selections of content to copy based on what is believed to be relevant.	*I mostly did chunks of [the vignette]. I usually just did the descriptive parts. Physician 3* *I might copy and paste the relevant part of the physical exam, but not the whole physical exam […] I try to narrow the search space so that I’m really getting what I want back. Physician 16* *I just had this assumption it would just overwhelm ChatGPT. So I didn’t copy and paste the whole case. I just copy and pasted more of the aspects of the information that would be more valuable to me in terms of affecting my decision making. Physician 15*
Summarizer	Makes assessment of relevant or important information from the vignette and summarizes the information in their own words to enter into the chatbot.	*I only put the relevant information, or at least what looked like the relevant information to me, from a case. I would summarize everything in there. Physician 16* *I more often tend to just summarize the pertinent findings. […] I feel like just convenience for myself, and also trying to reduce the amount of noise for ChatGPT. I don’t want it going down rabbit holes. Physician 19* *Another time I tried writing the case with the points that I thought were pertinent positives. I tried to distill it to, “All right, these are the pertinent positives.” […] I don’t want to feel like I’m outsourcing all of my thinking and literally just copy-pasting. Physician 17*
Searcher	Uses the chatbot like an internet search with basic queries; might include stringing together multiple concepts to ask a question, but limited to no more than one sentence.	*There were specific questions that I typed in the ChatGPT, because I needed the answer to come up with the three differential diagnoses. Physician 8* *I wanted to use it more as a tool to supplement me if I needed it. And that’s why I only asked it,* “*I’m thinking this, this is what I’m going to put in.*” *[…] I wanted to see if the data supported that and not have a computer give me a diagnosis initially. Physician 2* *I tried a couple more generic questions and the responses I got back were I think too vague to be helpful. […] I have a hard time knowing how to communicate what I need out of ChatGPT to give me back something useful. Physician 6*

### Physicians’ experiences using the chatbot by type

Physicians were asked to describe their rationale for inputting content into the chatbot and their perceptions of the subsequent outputs. These are explored below, organized by the approach taken for each case. Of note, nine interviewed physicians described trying more than one approach, as opposed to consistently using one strategy across all three cases. They described trying different approaches either because they wanted to experiment with seeing what the chatbot would do with a different amount of content, or because they felt that some cases would be better approached with a different input strategy. Some physicians who had not used LLM chatbots before described “discovering” that they could copy and paste large amounts of content into the chatbot.

#### Copy-pasters

Fifteen interviewed physicians described copy-pasting the entire vignette for at least one case. One rationale given for utilizing copy-pasting was in how it was perceived as the “easiest” method, either in how copy-pasting took less time than typing out new text, or for how using the instructions embedded in the case (i.e., “list three possible diagnoses”) was simpler than reformatting a new query. Some physicians also described utilizing copy-pasting when they wanted a more robust response from the chatbot, based on perceptions that a more detailed input would produce a more detailed output. Copy-pasting for the diagnostic cases was generally found to produce a detailed differential that could be easily skimmed, and was described as particularly useful for idea generation or confirmation. One physician described this as a “shotgun” approach, where they felt that a comprehensive list of possible diagnoses was an efficient way to “get their brain working.” In contrast, two physicians thought the outputs were too verbose to be very helpful to their problem-solving process.

#### Selective copy-pasters

Six physicians described using selective copy-pasting for at least one case. Selective copy-pasting allowed physicians to quickly capture specific pieces of the vignette that were of interest. Physicians described two primary rationales: 1) to focus only on specific pieces of the vignette, such as the labs or symptom description, or 2) because they were unsure of how much content the chatbot could “handle” and assumed less content would avoid “overwhelming” it. The outputs were generally described as helpful, but three physicians who experimented with copy-pasting on subsequent cases noted that by comparison, selective copy-pasting was a slower process and seemed to produce less detailed outputs.

#### Summarizers

Four physicians described using summarizing for at least one clinical case. Similar to selective copy-pasting, the rationale was to create an input that captured details of interest while leaving out information perceived as irrelevant. In contrast to selective copy-pasting, writing out a summary was perceived as a more flexible approach as physicians could incorporate information across the vignette in their own words, focusing only on the information that they felt was most relevant to prevent the chatbot from “going down rabbit holes.” In contrast, one physician tried summarizing primarily because they disliked how copy-pasting felt too much like “outsourcing” all of their thinking to a chatbot. For this participant, the process of creating their own summary helped them feel more cognitively engaged in the problem-solving process. One limitation of summarizing was that information could sometimes be missed, which was noted by a physician who had also tried copy-pasting and felt that the chatbot picked up on aspects of the case that they had “glossed over” in their summary.

#### Searchers

Seven physicians described using searching by inputting brief queries, similar to how one would use a search engine, for at least one case. Not every physician had a specific rationale for utilizing searching—one physician was unsure, and presumed that it may be because it was how they were used to looking up information on the internet. Some physicians noticed that large inputs would create large outputs, and felt that a more specific query was the best method for getting a concise output that addressed only their question and did not contain irrelevant information that they would have to take time to read through. Searching was often used when the physician already had a clear idea of how they were going to answer the case, but only needed a few pieces of specific information to complete their answer. The same physician who described copy-pasting as a “shotgun” approach also tried searching, and described this as a “sniper approach” that was valuable when seeking an answer for a “targeted” or specific question. Most physicians felt that they were easily able to elicit useful outputs based on their inputs. However, two physicians experienced more difficulty in generating relevant outputs, finding it very challenging to design the right question for the chatbot.

### Input approach type and clinical decision-making performance

**Fig. 2:**
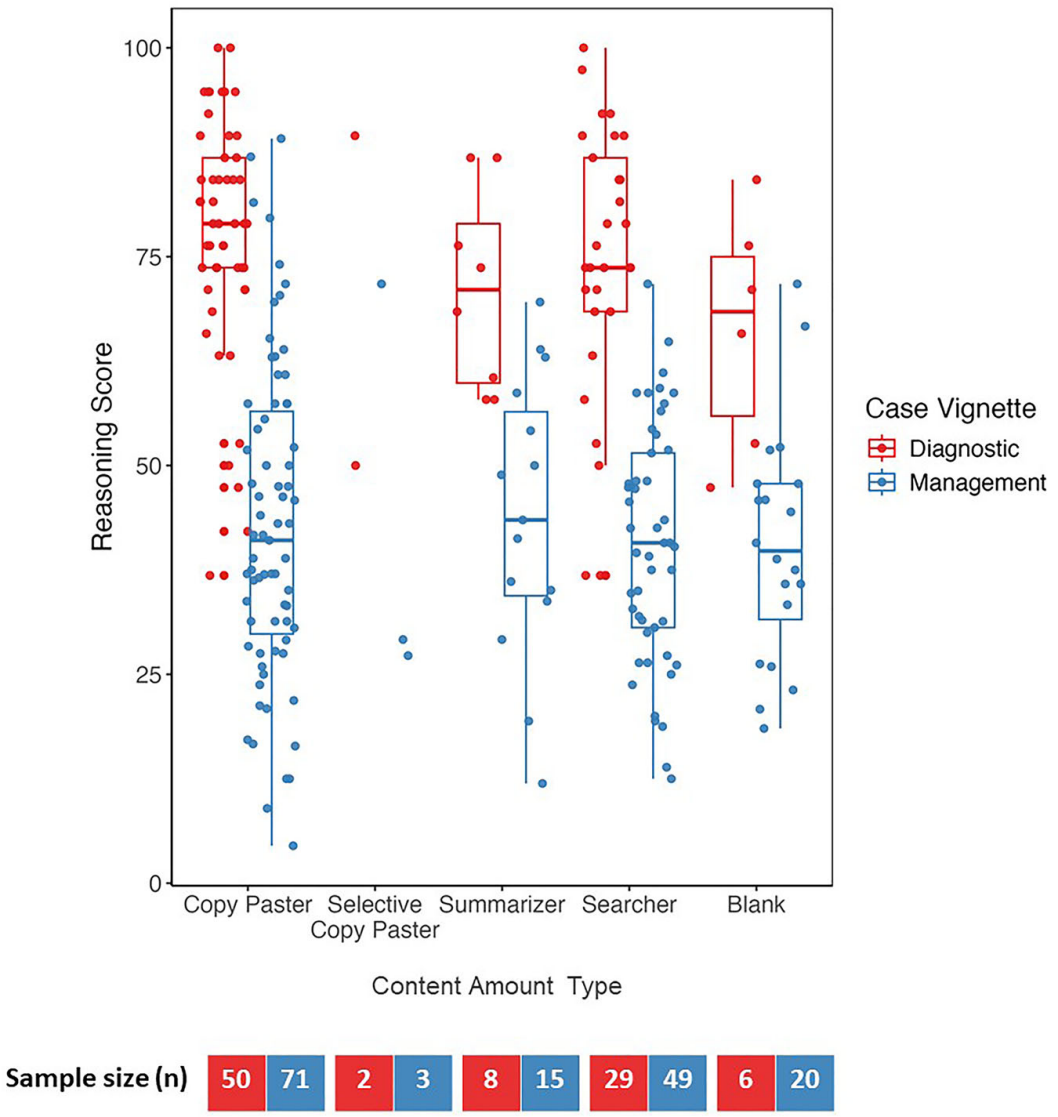
Clinical reasoning scores for diagnostic and management cases organized by content amount type. Blue indicates diagnostic cases and red indicates management cases. Analyzed using a linear mixed-effects model with random effects for participant and case to account for clustering by physician participant and case.

Figure 2 depicts the scores of participant cases from the intervention (access to the chatbot) arms of both RCTs, plotted by their type. The diagnostic RCT had 25 physician participants; 22 physicians completed a total of 95 cases that had both an accessible chatlog and a score, while the chat logs for 3 physicians could not be accessed. The management RCT had 46 physician participants; 42 physicians completed 158 cases with both an accessible chatlog and a score, while the chatlogs for 4 physicians could not be accessed. Cases that were started but not completed (e.g., had a chat log but were given a score of 0) were not included in the analysis. All four types were represented in both the diagnostic and management RCTs, but the most common approaches were copy-pasting or searching. In some instances, seen more often with the management cases, a case was worked through without using the chatbot at all (plotted in the “blank” column). For 4 of the 6 diagnostic cases, 1–2 participants did not use the chatbot on each case. Between 2 and 5 participants did not use the chatbot for the five management cases. No single type was associated with scoring higher on the clinical cases, for both diagnostic and management cases (see Supplementary Information for additional results from the model).

## Discussion

In this study, we developed a typology of content input styles that describe how physicians interacted with an LLM chatbot and explored if inputs based on content amount were associated with improved clinical reasoning. Though we hypothesized that the LLM chatbot may have performed better in the previous RCT because it was provided with the entire case vignette, physicians who used the copy and paste technique for their own clinical reasoning did not perform better than those who used other strategies. While content input amount may influence outputs, it may be that it is the ways in which physicians filter and use the information for their clinical reasoning that may ultimately influence the utility of AI for clinical decision making. As LLM chatbots grow in popularity and demonstrate greater abilities in working through clinical cases, this study adds to our understanding of how physicians approach using LLM chatbots and what may be needed to effectively collaborate with chatbots in clinical settings.

Our typology identified that physicians utilized four distinct “content amount” input styles (copy-pasting, selective copy-pasting, summarizing, and searching) when entering information into the chatbot. While no single style was associated with higher scores in clinical reasoning, copy-pasting and searching were the approaches utilized most. Previous studies of lay people’s use of chatbots for working through word problems have also reported frequent use of either copy-pasting or searching, where copy-pasting was often selected when users perceived copying as easier, while searching was often selected due to prevailing mental models of search engines^16,17^. Some physicians who used searching described aiming for more narrow outputs, whereas some physicians who used copy-pasting described aiming for more robust outputs. The literature on clinical problem-solving has highlighted how experienced physicians can vary greatly on the amount or type of data needed to work through the same clinical cases, which may be due to personal problem-solving styles or the uniqueness of their knowledge bases^18,19^. This typology elucidates the different intuitions and intentions that physicians may have when interacting with an LLM chatbot in the context of clinical problem-solving, which may be useful for various aspects of implementation. Training on LLM chatbot usage can be tailored to physicians’ different problem-solving styles or use cases, or chatbot developers could design LLM chatbots that are aligned with how physicians already approach using digital tools for clinical problem-solving. While LLM chatbots are increasingly being integrated with the Electronic Health Record (EHR)^20^, which represents a different use case compared to using LLMs in external applications, the use of LLMs in external applications will likely remain the norm in the interim.

Previous research has highlighted the significant potential that LLM chatbots may have in supporting clinical decision-making, such as through retrieving medical knowledge and generating differential diagnoses and treatment plans^21^. However, whether these tools make physicians feel more or less efficient than usual resources will be another important consideration for implementation. Physicians described various perceived inefficiencies when using an LLM chatbot, such as in struggling to come up with queries that generated a helpful response, or in generating too-long outputs when seeking more targeted information. Interviewed physicians also generally disliked the GPT-4 outputs for management cases—describing outputs as too broad or unable to consider patient- and setting-specific nuances—and reported relying more on their own clinical experience to work through these cases, which may be one reason why the chatbot was not utilized as much for the management cases^22^. Barmen et al. have argued that to support LLM users, there should be guidelines clarifying which tasks LLM chatbots can and can’t do well, which tasks require additional refinement, “context-specific heuristics,” and how to streamline interactions to support efficiency^23^. It will be important to continue to disseminate trainings and information on the different available LLM chatbot tools and their abilities, the strengths and limitations in using LLM chatbots for different clinical tasks, and how to best tailor LLM chatbot use to a case, patient, setting, or personal problem-solving style to support physicians in using these tools.

Our study was novel for seeking to understand how physicians working through clinical cases would intuitively approach selecting inputs for chatbots. Though the “prompt style” axis was not included in the final typology, asking the chatbot to “do something” with the content provided was still highly utilized by physicians, who used a range of different input styles both across and within cases, with some even describing having a “conversation” with the chatbot and asking it to provide reasoning. The results of our analysis of clinical case score by content amount type showed both a range of scores within each type, and also that no single type performed better than others. Research continues to test the effectiveness of different prompting techniques in the context of clinical problem-solving^6,24^. Guides for medical professionals have identified various best practices for prompt engineering, such as assigning a role, providing context, requesting examples, giving instructions, or specifying the desired output^9,25^. It may be the case that structured prompt engineering techniques such as these have more impact on the quality of the final output than the amount of content provided alone. In addition, there are many other variables that could impact the effectiveness of using an LLM chatbot for clinical problem-solving, such as, the nature of the problem-solving task, different LLM capabilities (e.g. multi-turn usage, ability to ask it to summarize its own outputs or asking it for its reasoning), or the level of cognitive engagement on the part of the physician. It will be important for future research to continue to examine prompt engineering strategies and effectiveness in generating accurate, useful outputs when using chatbots for clinical cases, as well as how best to educate physicians in their use. Since our results show that organic input styles by physicians can be quite variable, we suggest that future research first test prompting styles in controlled settings to identify which strategies are most effective before testing their effectiveness with real-world users.

All cases were based on real patient encounters, but did not involve real patients and were presented as vignettes. Participants from the two RCTs also had a time limit for completing the clinical cases. Participants may have approached information gathering and problem-solving in a real-world scenario differently, yet these mock cases are still informative, helping us understand how physicians might approach using a LLM chatbot for problem-solving. This study also involved a large number of resident trainees, and thus, may not be representative of the general physician population, but are still an important perspective for representing the next generation of practicing physicians trained in contemporary medical practices. This study mostly involved practitioners from inpatient and academic settings which also may not be representative of the general physician population—the experiences of practitioners in outpatient and community settings should be explored in future studies. There is high risk of confounding since strategy choice was not random and participants likely chose specific strategies based on factors related to case difficulty and their own knowledge in the clinical content area. We did not examine the chat log outputs for accuracy or quantity of content and so could not assess the variability in the chatbot responses, though LLMs are known to provide different responses to the same question.

Physician usage and understanding of LLM chatbots is variable, from copy-pasting entire clinical cases to treating chatbot systems similarly to search engines with brief queries, however, amount of content input into LLM chatbots was not related to physician performance on clinical reasoning tasks. Purposeful training and support is needed to acclimate physicians to learn how to effectively use emerging AI technologies to realize their potential for supporting safe and effective medical decision-making in practice.

## Methods

This study was performed in accordance with the Declaration of Helsinki and underwent an exempt review by the Stanford Institutional Review Board, eProtocol #73932, and Beth Israel Deaconess Medical Center, Protocol #2024P000074. Informed consent was obtained from all participants. No AI tools were used for analysis or manuscript writing.

### Physician interviews

We first conducted interviews with licensed physicians practicing in internal, family, or emergency medicine in the United States who were recruited by email from two previously conducted RCTs^13,15^ and by snowball sampling. Participating in the RCTs was not a prerequisite for participation. Interested participants signed up for a 30–60 min interview by either Google form or an email to the interviewer. Participants were offered an incentive of either $150 (for residents) or $199 (for attendings). For the purposes of the interview, participants were first sent a sample of three clinical case vignettes (two diagnostic and one management) used in the prior RCTs via Qualtrics survey and were instructed to complete the cases using GPT-4 (see Supplementary Information). The diagnostic cases asked participants to present their top three differential diagnoses, data for and against each, and up to three suggested next steps in the diagnostic evaluation. The management case asked participants to describe the next appropriate step for management decisions, such as medication choices or conversations with patients and family. We then conducted qualitative semi-structured interviews with the participants to explore their experiences of using GPT-4 for the three cases; the surveys were not graded or analyzed. The interview topic guide (see Supplementary Information) covered the following topics: prior experience using AI tools, experiences using the chatbot to complete the mock cases, how they entered information into the chatbot, rationales for what they entered into the chatbot, and how they used the chatbot’s outputs in their decision-making. Interviews were conducted over Zoom by either LMH, an experienced qualitative health services researcher, or HK, a physician with training in conducting qualitative interviews. Interviews were recorded with permission and transcribed for analysis. Interviewers met regularly during data collection to discuss impressions, and continued conducting interviews until they agreed that no new themes arose.

Interview transcripts were imported into NVivo and analyzed using the Framework Method^26,27^. The Framework Method is a structured approach to analysis using both deductive and inductive coding that can be utilized to develop a typology^28,29^. All qualitative analysts (LMH, HK, RS) familiarized themselves with the interviews by listening to the recordings or reading the transcripts. Transcripts were coded by RS and initial coding was reviewed by LMH, with emergent codes reviewed by the entire qualitative team at monthly meetings and refined through discussion. After coding, a framework matrix was created in NVivo with participants represented in rows, and code groupings presented as columns. Data was then charted into the framework matrix by summarizing the data within each cell to facilitate the identification of patterns of GPT-4 use, experiences using GPT-4 to work through the clinical cases, and participants’ rationales for different input strategies. For developing the typology, RS and LMH independently analyzed the described characteristics of inputs, as summarized in the framework matrix, to develop initial categories of use, and then met to compare results and check for alignment before finalizing the typology.

### LLM chat log coding

The recorded interactions between the intervention arm participants and the chatbot (chat logs) were downloaded from the two RCTs^13,15^. In the intervention arms of each RCT, physicians were given access to GPT-4 (provided by the researchers) in addition to usual clinical care resources (e.g. UptoDate, Lexicomp) to complete clinical vignettes^13,15^. Physicians were not required to use the chatbot on every case. Participants had a one-hour time limit and were instructed to prioritize quality of responses over completing all cases. In the diagnostic RCT, participants completed up to 6 cases^13^, and in the management RCT, participants completed up to 5 cases^15^.

Three physician coders independently applied the definitions of input approach typologies to the chat logs, coding each completed clinical case to one of the input approach types in the framework, or as a new type if none applied. Cases, not participants, were coded to an approach type because physician participants may have used different approaches for different cases. After each chat log was coded by at least two physicians, the coding was reconciled. Any discrepancies in coding were discussed by the coders and the third physician acted as adjudicator as needed. If multiple approaches were used on a single case, coders reached agreement on the highest amount of content input and coded the case to that type. Following chat log coding, the coders met to discuss coding challenges and check if there were any new approach types identified in the chat logs to further refine the typology.

### Clinical case scores and input approach type

In the two previous RCTs, grading rubrics were developed for each study via pilot data and iterative feedback from graders and pilot participants (see Supplementary Information for examples). Each case was scored by two blinded physician graders. When there was disagreement (predefined as greater than 10%), they met to discuss and seek consensus^13,15^. We then used a linear mixed-effects model to assess how each input approach type related to performance on diagnostic and management reasoning tasks. Linear mixed-effects models were applied to assess the difference in performance by typology type, with normality assumptions verified. Random effects for participant and case were included in the model to account for clustering of scores by physician participant and case. A term for study, diagnostic, or management reasoning, was also included. Analyses were performed in R version 4.4.0 (R Project for Statistical Computing) using the Ime4 package.

## Supplementary information

41746_2025_2184_MOESM1_ESM.

## Data Availability

The qualitative interview data collected during this study are not publicly published to respect the privacy of the participants but are available from the corresponding author on reasonable request. The RCT data used in this study was not made publicly available but is available from the studies’ authors on reasonable request.
