# Supplementary material for: A typology of physician input approaches to using AI chatbots for clinical decision-making
Source: NPJ Digit Med. 2025 Dec 5;9:14. doi: 10.1038/s41746-025-02184-y (PMC12780094; doi:10.1038/s41746-025-02184-y)
Supplement: Supplementary file 1 — 41746_2025_2184_MOESM1_ESM. [file 41746_2025_2184_MOESM1_ESM.pdf]

I. Qualtrics Survey

Intro Block

Thank you for participating in our research study. Throughout the survey, you will work on **2 diagnostic cases and 1 management case**, presented in a randomized order.

Before we proceed, please kindly provide your information.

First and Last Initials followed by home street number
Ex: if your name was Tom Ford and you live at 1111 Main Street, you would put “TF1111”).

Email

Specialty

- ○ Internal Medicine

- Family Medicine
- Emergency Medicine

How many years of experience in medicine do you have, including residency training?
Ex: if you have completed 3 years of residency training and have been an attending for two years, then you would enter "5".

Prior Experience with Generative AI / ChatGPT

- I've never used it before
- I've used it once ever
- I use it rarely (less than once per month)
- I use it occasionally (more than once per month but less than weekly)
- I use it frequently (weekly or more)

Diagnostic Case Example

Example:

In this study, you are going to read diagnostic cases and complete three discrete sections for each case: structured reasoning, your final diagnosis, and the next diagnostic steps. Here is an example case.

Example Case

HISTORY OF PRESENT ILLNESS

A 24-year-old woman who is sixteen weeks pregnant is seen in the office twelve hours after the onset of abdominal pain. The pain is severe, steady, and located in the epigastrium. She vomits several times during the interview. She does not have diarrhea. Her pregnancy has been uneventful. She has no history of gastrointestinal disease. Her only medications are a daily multivitamin

PHYSICAL EXAMINATION

On examination the blood pressure is 110/60 mmHg and the pulse 88/min. and regular. The temperature is 38.2°C. Mild jaundice is present, but there are no spider angiomas. The eyes and nose are normal, but the tympanic membranes show no light reflex. The cardiac PMI is normally placed, and there is a soft systolic murmur along the left sternal border. The second heart sound splits normally. Severe epigastric tenderness is present and the bowel sounds are hypoactive. The liver is not palpable, but the spleen is two fingerbreadths below the left costal margin. The uterus is palpable 10 cm above the pubis.

LABORATORY EXAMINATION

Hematocrit 39%
WBC count 12,600/mm3
Platelets 185,000/mm3
Neutrophils 88%
Bands 3%
Lymphocytes 9%
Bilirubin (total) 4.6 g/dL
Alkaline phosphatase 300 U/L

ALT 120 U/L
AST 100 U/L
Albumin 3.2 g/dL
Total protein 6.5 g/dL
Prothrombin time, normal
Amylase 860 U/L
Lipase 1210 U/L
pH 5.6 (urinalysis)
RBCs 0/HPF (urinalysis)
WBCs 2-3/HPF (urinalysis)
Bacteria occasional/HPF (urinalysis)

RADIOLOGY
A plain film of the abdomen shows a normal bowel gas pattern.

Example Matrix (Answer)

PART 1: Structured Reasoning

	Diagnosis<br><br>List 3 possible diagnoses below	Supporting Factors<br><br>For each possible diagnosis listed, provide findings/risk factors supporting this hypothesis	Opposing Factors<br><br>For each possible diagnosis listed, provide findings opposing this hypothesis, or findings that were expected by not present
1	Gallstone pancreatitis	Elevated Lipase, Amylase, Bilirubin, AST, ALT, WBC, Temp, neutrophils, bands. Vomiting + epigastric pain.	No pain with eating. Young age for gallstones
2	Pregnancy Loss / Spontaneous Abortion	Pain (cramps), known pregnancy	No vaginal bleeding. Many unexplained lab abnormalities including elevated lipase, AST/ALT
3	HELLP Syndrome	Pregnancy, transaminitis<br>Abdominal pain	Early in pregnancy<br>No hypertension or proteinuria to suggest preeclampsia. No hemolysis or low platelets

Example Most likely Diagnosis

PART 2: Final diagnostic decision

Based upon your reasoning above, what is your final diagnosis?

Gallstone Pancreatitis

Click to write the question text

PART 3: Additional Steps

Name up to 3 additional steps that you would take in your diagnostic process

1	Ultrasound of the abdomen including right upper quadrant and uterus
2	Additional laboratory tests: fractionated bilirubin, Calcium, triglycerides
3	Ask about amount and pattern of alcohol intake as a risk factor for alcoholic pancreatitis.

Diagnostic Case 1

DIAGNOSTIC CASE 1 (3 Parts)

History Of Present Illness

A 76M comes to his PCP complaining of pain in his back and thighs for 2 weeks. He has no pain sitting or lying, but walking causes severe pain in his low back, buttocks and calves. He feels febrile and tired. He was told by the referring cardiologist that his recent tests results since the pain started showed a new anemic and azotemia. A few days before the onset of the pain he had undergone coronary angioplasty. Heparin was administered for 48 hours.

Past Medical History

Ischemic heart disease had first been diagnosed ten years earlier, at which time a coronary artery bypass procedure was done.

Physical Examination

VITALS: 99.6° F.; pulse was 94/min and regular; BP was 110/88 mmHg.
GEN: Well appearing
CARDS: There is a grade III/VI apical systolic murmur; lungs were clear to auscultation.
PULM: Lungs are clear to auscultation bilaterally, no wheezing, or consolidations noted
ABD: Soft, non-tender to palpation
MSK: He does not have tenderness of his spine or pelvis. Spinal mobility is normal, as is the mobility of his hips. Standing is painless; however, pain is experienced in his low back, buttocks and calves within a minute of feeble running in place. The pain disappears shortly after exercise is discontinued.
EXT: Peripheral pulses were symmetrically reduced, but palpable. The neurological examination was normal.
SKIN: Patient has a purple, red, lacy rash over his low back and buttocks.

Laboratory

WBC of 11.5 x 103 cells /μL; differential of 64% segs, 20% lymphocytes, 3% monocytes, 12% eosinophils and 1% basophil. The hematocrit was 28% and the platelet count was 315 x 103 /μL. The erythrocyte sedimentation rate was 99 mm/h. Urinalysis was normal except for 2+ proteinuria. Serum creatinine was 4.0 mg/dL; sodium was 145 mEq/L; potassium 4.0 mEq/L; chloride 105 mEq/L. SGOT was 27 U/L; GGT was 90 U/L; alkaline phosphatase was 153 U/L.

PART 1: Structured Reasoning

	Diagnosis<br><br>List 3 possible diagnoses below	Support diagnosis<br><br>For each possible diagnosis listed, provide findings/risk factors supporting this hypothesis	Opposing diagnosis<br><br>For each possible diagnosis listed, provide findings opposing this hypothesis, or findings that were expected by not present
1			
2			
3			

PART 2: Final diagnostic decision

Based upon your reasoning above, what is your final diagnosis?

PART 3: Additional Steps

Name up to 3 additional steps that you would take in your diagnostic process

1

2

3

Diagnosis Case 2

DIAGNOSTIC CASE 2 (3 Parts)

History of Present Illness

A previously healthy 64M presents with a six month history of fatigue, 30 pound weight loss, and worsening skin rash. He states, “I have no energy.” In the past month, he suddenly cannot climb stairs or rise from a chair without assistance. The rash, which began on his elbows and knees, has spread to his hands and face. He has minimal appetite, but feels constantly bloated and notes constipation He denies pain, hoarseness, or dysphagia. His only medication is topical steroids recently provided for his skin rash.

Past History

Until this illness he developed the skin rash he had never seen a physician.

Systems Review

His system review is negative except for the present illness.

Family History

Father died at 64 with colon cancer. Mother died at 60 from a stroke. One brother died at 66 with renal cell carcinoma.

Social History
He smokes one pack of cigarettes per day. He drinks two "six-packs" most weekends.

Physical Examination
VITALS: His blood pressure is 140/90 mmHg, his pulse is 80/min., and his respiratory rate is 15/min.
GEN: The patient is a thin, pale, white male in no acute distress.
CARD: Normal rate and rhythm and no murmurs, gallops, rubs
PULM: Lungs are clear to auscultation bilaterally
ABD: The abdomen is distended and is tympanitic to percussion. Bowel sounds are normal. The rectal exam reveals no masses, but the stool is positive for occult blood
GU: The GU exam is normal.
EXT: No pitting edema
Skin: scaly, erythematous eruption involving the dorsum of the elbows, knees, and interphalangeal and metacarpophalangeal joints. A blue-red rash is present on his face
Neuro: Neurological examination revealed symmetrical proximal quadriceps muscle weakness of the iliopsoas and quadriceps

Laboratory and Other Procedures
WBC is 12,400/µL with 82% PMN's, 16% lymphocytes and 2% monocytes. Hemoglobin is 9.5 g/dL, hematocrit 30% and MCV is 69 fL. Platelets are 660,000/µL. The erythrocyte sedimentation rate is 90 mm/hr. Chemistry panel: Normal except for the Creatine phosphokinase which is 440 IU (nl 47-220).

PART 1: Structured Reasoning

	Diagnosis<br><br>List 3 possible diagnoses below	Support diagnosis<br><br>For each possible diagnosis listed, provide findings/risk factors supporting this hypothesis	Opposing diagnosis<br><br>For each possible diagnosis listed, provide findings opposing this hypothesis, or findings that were expected by not present
1			
2			
3			

PART 2: Final diagnostic decision

Based upon your reasoning above, what is your final diagnosis?

PART 3: Additional Steps

Name up to 3 additional steps that you would take in your diagnostic process

1

2

3

Management Case 1

MANAGEMENT CASE (5 Questions)
HISTORY OF PRESENT ILLNESS:
A 72 year-old Vietnamese-speaking woman with HLD, HTN, CKD stage II (baseline creatinine 1.3), cholelithiasis is admitted to the hospital for severe gallstone pancreatitis.

PMH/PSH:
Hyperlipidemia
Hypertension
Cholelithiasis
CKD stage II
History of positive PPD
Right knee replacement age 65

FHx:
Heart disease
Diabetes

SHx:
Former smoker
Drinks 1-2 drinks of ETOH daily

Lives with her son and daughter in law
Widowed
Used to work managing in a textile factory

Pre-admission medication list:
-Atorvastatin 40mg qhs
-Lisinopril 5mg qD
-Ibuprofen 400-800mg qD PRN for joint pain

PHYSICAL EXAM:
T 101.3, HR 112, BP 98/52, O2 sat 93% RA, RR 24
Gen: Alert, oriented, in NAD, appears very fatigued
HEENT: mild scleral icterus
CV: tachycardic, regular rhythm, no m/r/g
Lungs: crackles in the bases bilaterally
Abdomen: soft, mildly distended, TTP in the RUQ/peri-umbilical areas
Ext: no LE edema
MSK: no joint swelling

LABORATORY AND OTHER PROCEDURES:
CBC showed leukocytosis to 17.3K, decreased Hgb to 10.2, and elevated platelet count to 345K.
BMP showed elevated creatinine to 1.8, elevated BUN to 45.
LFTs show elevated AP to 102, elevated Tbili to 3.2.
Lipase is elevated to 603.
CT A/P shows peripancreatic stranding consistent with pancreatitis, cholelithiasis, and intrahepatic biliary ductal dilatation.

CASE CONTINUATION:
Her course is complicated by severe sepsis and ARDS requiring 6L supplemental O2, for which she is transferred to the ICU. She is treated with broad spectrum antibiotics and frequent electrolyte repletion. A chest x-ray obtained to evaluate for pneumonia shows a lung nodule, bilateral

interstitial markings but no consolidation. After transfer out of the ICU, she has a follow up CT with contrast that showed improvement of the interstitial edema and reconfirms a single 2.5 cm nodule; the radiology report suggests either biopsy or repeat imaging in three months. Because she immigrated from Vietnam and has a history of positive PPD, the infectious disease team is consulted and recommends ruling her out for tuberculosis. Three acid-fast bacilli smears are sent, as is a nucleic acid amplification test, and all are negative for tuberculosis.

Her course is further complicated by the development of a DVT, which requires a heparin drip with transition to apixaban, as well as respiratory failure requiring intubation. Despite a prolonged hospital course – she is an inpatient for 57 days – she gradually recovers. She is severely deconditioned, and rehabilitation is recommended.

On the day of discharge – 54 days after her lung nodule is discovered – she is preparing for discharge. Her lung nodule has long ago dropped off her progress notes, but it is again noted on a review of imaging prior to discharge.

DISCHARGE MEDICATION LIST:
-Atorvastatin 40 mg qhs
-Lisinopril 5 mg qD on HOLD until patient follows up with PCP
-Ibuprofen 400-800 mg qD PRN for joint pain on HOLD given mild AKI on CKD during hospitalization
-Acetaminophen 1000 mg q8h PRN for pain
-Potassium chloride 20mEq daily
-Apixaban 5mg BID

DISCHARGE PHYSICAL EXAM:
T 97.3F, HR 85, BP 142/75, O2 sat 95% RA, RR 18
Gen: Alert, oriented, in NAD, appears weak
HEENT: no scleral icterus, no oral lesions

CV: RRR, no m/r/g
Lungs: CTAB, no c/r/w
Abdomen: soft, non-distended, non-tender to palpation
Ext: no LE edema
MSK: no joint swelling

DISCHARGE LABS:
CBC:
WBC 10.5
Hgb 8.3
Plt 473

BMP:
Sodium 137
K 3.4
Chloride 104
Bicarb 21
BUN 26
Creatinine 1.5

Question 1

Based on the information you have at this time, what is the differential diagnosis for this incidental lung nodule?

Question 2

What additional information (from patient or medical record) could you non-invasively obtain that would help further hone the differential diagnosis for this lung nodule?

Question 3

This patient is being planned for discharge today and is expecting to leave today to go to rehab. What factors would influence your decision on keeping the patient in the hospital or deferring to the outpatient setting for additional work up?

Question 4

If you were to defer the workup to the outpatient setting, what would be your process?

Question 5

If you were to keep the patient in the hospital for additional investigations, at what point in those investigations would you discharge the patient?

Powered by Qualtrics

II. Topic Guide

Introduction:

This is a multi-center qualitative study assessing physicians' views on the use of artificial intelligence in clinical practice. We will be performing a series of interviews with around 20 doctors. All information we gather will be anonymized and securely stored.

Do you have any questions for me before we begin?

Is it OK to record?

Main questions	Prompts
**Background**: First, I'd like to start with some background information.	
Could you tell me a bit about yourself, like your clinical interests and how long<br>you've been in practice?	
What do you enjoy most about being a doctor?	
Do you have any previous experience using AI for either clinical or personal use? [ChatGPT, Dali, Claude, BingAI]	If yes, probe for brief details.<br>Have you had any training in creating prompts for working with AI?
**Views on GPT**: Thanks for that background information. I now want to talk about your experience<br>using GPT for clinical decision making, as you did in the survey with clinical questions for this study.	
How did it go using GPT for clinical decision making?	How did you input information or questions to GPT?<br>Why did you input information in that way?/ Why did you take the approach you did to using GPT?<br>What did you like about GPT?<br>What didn't you like?
How confident are you that you got to the right diagnosis or solution?	Was GPT better for diagnostic or management decision<br>making, or equal? Why?
How efficiently do you feel you got to the<br>diagnosis/solution you did?	Did GPT slow down or speed up your clinical decision making?
In what ways did GPT change your usual process for clinical decision making?	What about working with Chat GPT was useful? Did working with Chat GPT feel like consulting a dictionary, talking to a colleague,<br>or something else entirely?<br>Did it prompt you to take a different approach to clinical decision making?<br>Are there instances where ChatGPT surprised you with a unique perspective or solution to a diagnostic problem?
How trustworthy do you feel the outputs of GPT were?	In what ways could the trustworthiness of it be improved?<br>Were there specific situations where you disagreed with the AI's suggestions?<br>Did your trust in ChatGPT's suggestions evolve over the course of the study? How/Why?
**Integration into routine use**: My last set of questions are about how GPT or other AI tools might be	

integrated into routine use for clinical decision making.	
What do you think it would take to integrate AI into routine clinical decision making?	What things might help facilitate its use?<br>What do you see as the barriers to use?
Is there anything that concerns you about integrating Chat GPT or similar AI into routine clinical decision making?	E.g. ethics, patient perceptions, governance.
When there is a new innovation in clinical practice, do you like to be the first person to try it out, or do you prefer to adopt it later? (Innovator, early adopter, early majority, late majority, laggards)	
Is there anything else you'd like to add about the use of AI for clinical decision making that I haven't already asked about?	

III. Example Diagnostic Case and Grading Rubric

SUPPLEMENTARY TABLE 1: EXAMPLE DIAGNOSTIC VIGNETTE

History Of Present Illness

A 76M comes to his PCP complaining of pain in his back and thighs for 2 weeks. He has no pain sitting or lying, but walking causes severe pain in his low back, buttocks and calves. He feels febrile and tired. He was told by the referring cardiologist that his recent tests results since the pain started showed a new anemia and azotemia. A few days before the onset of the pain he had undergone coronary angioplasty. Heparin was administered for 48 hours.

Past Medical History

Ischemic heart disease had first been diagnosed ten years earlier, at which time a coronary artery bypass procedure was done.

Physical Examination

VITALS: 99.6° F.; pulse was 94/min and regular; BP was 110/88 mmHg.

GEN: Well appearing

CARDS: There is a grade III/VI apical systolic murmur.

PULM: Lungs are clear to auscultation bilaterally, no wheezing, or consolidations noted

ABD: Soft, non-tender to palpation

MSK: He does not have tenderness of his spine or pelvis. Spinal mobility is normal, as is the mobility of his hips. Standing is painless; however, pain is experienced in his low back, buttocks and calves within a minute of feeble running in place. The pain disappears shortly after exercise is discontinued.

EXT: Peripheral pulses were symmetrically reduced, but palpable. The neurological examination was normal.

SKIN: Patient has a purple, red, lacy rash over his low back and buttocks.

Laboratory

WBC of 11.5 x 103 cells /µL; differential of 64% segs, 20% lymphocytes, 3% monocytes, 12% eosinophils and 1% basophil. The hematocrit was 28% and the platelet count was 315 x 103 /µL. The erythrocyte sedimentation rate was 99 mm/h. Urinalysis was normal except for 2+ proteinuria. Serum creatinine was 4.0 mg/dL; sodium was 145 mEq/L; potassium 4.0 mEq/L; chloride 105 mEq/L. SGOT was 27 U/L; GGT was 90 U/L; alkaline phosphatase was 153 U/L.

Part 1 – Structured Reasoning Grid

	Diagnosis<br>List 3 possible diagnoses below	Support diagnosis<br>For each possible diagnosis listed, provide findings/risk factors supporting this hypothesis	Opposing diagnosis<br>For each possible diagnosis listed, provide findings opposing this hypothesis, or findings that were expected by not present
1			
2			
3			

Part 2 – Final diagnostic decision

**Question**	Based upon your reasoning above, what is your final diagnosis?

Part 3 – Additional Steps

**Question**	Name up to 3 additional steps that you would take in your diagnostic process

SUPPLEMENTARY TABLE 2: EXAMPLE OF HIGH-SCORING RESPONSE			
**Part 1 – Structured Reasoning**			
**Questions**		**High Scoring Example**	**Scores**
**Question 1**	Diagnosis - List 3 Possible Diagnosis	Cholesterol embolism	1/1
		Acute interstitial nephritis	1/1
		Peripheral arterial disease	1/1
**Question 2**	Support Diagnosis - For each possible diagnosis listed, provide findings/risk factors supporting this hypothesis	Cholesterol embolism - Recent PCI, multi organ involvement (peripheral vasculature, renal, skin), rash, renal failure, eosinophilia, known CAD	2/2
		Acute interstitial nephritis - Renal failure, rash, +/- anemia, proteinuria, all after intervention with many medications (including ASA)	2/2
		Peripheral arterial disease - Leg pain associated with ambulation, improves with rest	2/2
**Question 3**	Opposing Diagnosis - For each possible diagnosis listed, provide findings opposing this hypothesis, or findings that were expected by not present	Cholesterol embolism - Would expect leg pain to be at rest (acute occlusion rather than more of a stable angina type picture), not sure would present with fever, anemia not necessarily explained either	2/2

		Acute interstitial nephritis - Does not explain leg pain, did not receive classic causative medications / no other risk factors	2/2
		Peripheral arterial disease - Would not expect nearly any of the lab abnormalities, unlikely to present this acutely	2/2
**Part 2 – Final diagnostic decision**			
**Question**	Based upon your reasoning above, what is your final diagnosis?	Cholesterol embolism	2/2
**Part 3 – Additional Steps**			
**Question**	Name up to 3 additional steps that you would take in your diagnostic process	Arterial ultrasounds of bilateral lower extremities / ABI, analyze urine for casts / eosinophils, nephrology consultation and consideration of renal biopsy	2/2
**Total score**			19/19

In Table S2 is an example of a high-scoring response. The participant has three plausible diagnoses, with entirely correct and thorough answers for factors both supporting and opposing their diagnoses, as well as an entirely correct final diagnosis (cholesterol embolism).

IV. Example Management Case and Grading Rubric

CLINICAL VIGNETTE (PART 1):

A 72 year-old woman with a history of GERD, type 2 diabetes, and poorly controlled hypertension presents with fevers, chills, and abdominal pain. A CT of her abdomen shows perforated cholecystitis and peritonitis. She is started on broad spectrum antibiotics and taken for an urgent open cholecystectomy. The procedure went well, and it was without any immediate complications.

On post-operative day #1, you are paged by nursing after she was noted to have a brief run of asymptomatic atrial fibrillation picked up on telemetry. On detailed review of her telemetry data, in the last 24 hours she has experienced 3 brief episodes of atrial fibrillation, each lasting 15-20 seconds.

From the perspective of her cholecystitis and peritonitis, she is clinically improving, has had no fevers or chills since the surgery, and she is tolerating a liquid diet. Her blood pressure and heart rate are currently normal. Her labs are notable only for a downtrending leukocytosis. Both a preoperative ECG and a current ECG show normal sinus rhythm without any significant abnormalities. She has no prior echocardiogram. She is currently on no anticoagulation or antiplatelet medications.

QUESTION 1:

In addition to continuing telemetry monitoring, would you recommend any additional monitoring, testing, or treatment at this time? If so, what?

This question has a total of <u>4 points</u>.

Prophylactic anticoagulation to prevent VTE/DVT (e.g. enoxaparin 40mg daily) – Credit for either correct labeling of the intervention, or correct med + dose combination	1 point
TSH	1 point
Inclusion of up to 2 of the following:<br>● Echocardiogram<br>● Electrolyte assessment and/or repletion<br>● Evaluation for additional triggers (e.g. pain, constipation, etc…)	2 points max (1 point each)

CLINICAL VIGNETTE (PART 2):

There are no episodes of atrial fibrillation on either post-operative days #2 or #3. However, on post-operative day #4, the day prior to anticipated discharge, the patient again develops another episode on telemetry that is confirmed by ECG. This episode is also completely asymptomatic. It lasts two hours, during which the ventricular rate ranges from 70 to 105 beats/min. Her blood pressure is not affected. It spontaneously converts back to normal sinus rhythm.

QUESTION 2

Would you recommend to this patient that she be started on therapeutic anticoagulation?

What factors would influence your decision?

This question has a total of 9 points.

Decision on anticoagulation

Yes, start therapeutic anticoagulation	2 points

Factors which influence the decision

Patient preference and/or risk tolerance	2 points
• CHA2DS2-VASc or CHADS2 score<br>• Listing only 1-2 component of these scoring systems separately, without mentioning the score itself	2 points<br>1 point
Inclusion of up to 3 of the following 5 factors:<br>• HAS-BLED score<br>• Duration of a-fib episodes<br>• # of post-op episodes<br>• Information potentially obtained from an echo<br>• A-fib burden (i.e. % of time spent in a-fib)	3 points max (1 point for each)

QUESTION 3:

If the decision was made to start the patient on therapeutic anticoagulation on hospital day #4 (i.e. immediately after the asymptomatic, 2 hour long episode of a-fib), what would be the best choice for anticoagulation?

This question has a total of 3 points.

Apixaban, rivaroxaban, edoxaban, dabigatran, therapeutic enoxaparin	3 points
Unfractionated heparin gtt	1 point

QUESTION 4:

This question has a total of 7 points.

The patient is about to be discharged. Would you discharge her with an ambulatory ECG monitor?

Yes	1 point

If so, what type of monitor would you choose?

Holter and/or Ziopatch (i.e. “14 day Holter”)	1 point

What factors should be considered in the choice of monitor for this patient?

Inclusion of up to 2 of the following:	2 points max (1 point each)

• Cost of monitor<br>• Ease of insurance approval<br>• Duration of monitoring period<br>• Likelihood of the patient experiencing a lethal arrhythmia	

What would you do with the information from the monitor?

Determine anticoagulation plan (i.e. whether or not the patient requires anticoagulation, or the duration of anticoagulation)	1 point
Inclusion of up to 2 of the following:<br>• Determine if the patient requires rate control<br>• Determine if the patient requires rhythm control<br>• Determine if the patient requires more diagnostic testing	2 points max (1 point each)

V. Linear Mixed-Effects Model Additional Results

Model outputs:
Linear mixed model fit by REML ['lmerMod']
Formula: total_pct ~ Content + study + (1 | Case_n) + (1 | id)
Data: typo_full

REML criterion at convergence: 1986.1

SUPPLEMENTARY TABLE 3: Scaled Residuals				
**Min**	**1Q**	**Median**	**3Q**	**Max**
-3.1735	-0.4831	0.0794	0.6194	2.8401

SUPPLEMENTARY TABLE 4: Random Effects		
**Groups Name**	**Variance**	**Standard Deviation**
id (Intercept) (N=56)	61.23	7.825
Case_n (Intercept) (N=11)	75.92	8.713
Residual	117.81	10.854

SUPPLEMENTARY TABLE 5: Fixed effects		
	**Estimate**	**Std. Error**
Intercept (Copy-paster, Diagnostic Study)	77.0814	4.2069
Selective Copy Paster	2.6312	6.6074
Summarizer	0.1868	3.2319
Searcher	-2.4819	2.3848
Blank	-3.1089	2.9218
Management Study	-34.4841	5.7299

VI. Reporting Checklist

Good Reporting of A Mixed Methods Study (GRAMMS) checklist

Guideline	Section: page
Describe the justification for using a mixed methods approach to the research question	Introduction: pp. 3-4<br>Methods: pp. 13-15
Describe the design in terms of the purpose, priority and sequence of methods	Introduction: pp. 3-4<br>Methods: pp. 13-15
Describe each method in terms of sampling, data collection and analysis	Methods- under Physician Interviews, pp. 13-14; under LLM Chat Log Coding, pp. 14-15; and under Clinical Case Scores and Input Approach Type, p. 15.
Describe where integration has occurred, how it has occurred and who has participated in it	Methods- under Physician Interviews, pp. 13-14; under LLM Chat Log Coding, pp. 14-15; and under Clinical Case Scores and Input Approach Type, p. 15.
Describe any limitation of one method associated with the present of the other method	Discussion, pp. 11-12
Describe any insights gained from mixing or integrating methods	Discussion, pp. 9-12

Ref: O'Cathain A, Murphy E, Nicholl J. The quality of mixed methods studies in health services research. J Health Serv Res Policy. 2008;13: 92-98
